# Spectral Preprocessing for Clustering Time-Series Gene Expressions

**DOI:** 10.1155/2009/713248

**Published:** 2009-02-24

**Authors:** Wentao Zhao, Erchin Serpedin, Edward R Dougherty

**Affiliations:** 1 Electrical and Computer Engineering Department, Texas A&M University, College Station, TX 77843, USA; 2 Translational Genomics Research Institute, 400 North Fifth Street, Suite 1600, Phoenix, AZ 85004, USA

## Abstract

Based on gene expression profiles, genes can be partitioned into clusters, which might be associated with biological processes or functions, for example, cell cycle, circadian rhythm, and so forth. This paper proposes a novel clustering preprocessing strategy which combines clustering with spectral estimation techniques so that the time information present in time series gene expressions is fully exploited. By comparing the clustering results with a set of biologically annotated yeast cell-cycle genes, the proposed clustering strategy is corroborated to yield significantly different clusters from those created by the traditional expression-based schemes. The proposed technique is especially helpful in grouping genes participating in time-regulated processes.

## 1. Introduction

A cell is the basic unit of life, and each cell contains instructions necessary for its proper functioning. These instructions are encoded in the form of DNAs that are replicated and transmitted to its progeny when the cell divides. mRNAs are middle products in this process. They are transcribed from DNA segments (genes) and serve as the templates for protein translation. This conduit of information constitutes the *central dogma* of molecular biology. The fast evolving gene microarray technology has enabled simultaneous measurement of genome-wide gene expressions in terms of mRNA concentrations. There are two types of microarray data: time series and steady state. Time-series data are obtained by sequential measurements in temporal experiments, while steady-state data are produced by recording gene expressions from independent sources, for example, different individuals, tissues, experiments, and so forth. The high costs, ethical concerns, and implementation issues prevent from collecting large time-series data sets. Therefore, about 70% of the data sets are steady state [1], and most of time-series data sets contain only a few time points, in general less than 20 samples.

Based on microarray measurements, clustering methods have been exploited to partition genes into subsets. Members in each subset are assumed to share specific biological function or participate in the same molecular-level process. They are termed as coexpressed genes and are supposed to be located closely in the underlying genetic regulatory networks. Eisen et al. [2] applied the hierarchical clustering to partition yeast genes, Tamayo et al. [3] exploited the self-organizing map (SOM), and Tavazoie et al. [4] employed K-means clustering to group gene expressions and then search upstream DNA sequence motifs that contribute to the coexpression of genes. Besides the above mentioned successful applications, Zhou et al. [5] designed a clustering strategy by minimizing the mutual information between clusters, and bootstrap techniques were combined with heuristic search to solve the underlying optimization problem. Also, Giurcăneanu et al. [6] exploited the minimum description length (MDL) principle to determine the number of clusters. Whether technically advanced schemes represent better solutions for real biological data is still under debate. However, usually most of the schemes provide valuable alternatives and insights to each other. Therefore, it was recommended that several clustering schemes be performed to analyze the same real data set [7] so that the difference between clusterings would capture some patterns that otherwise would be neglected by running only one method.

A straightforward application of clustering schemes will cause the loss of temporal information inherent in the time-series measurements. This shortcoming has been noticed in literature. Ramoni et al. [8] designed a model-based Bayesian method to cluster the time-series data and specified the number of clusters intelligently, Tabus and Astola [9] proposed to fit the data by linear dynamic systems, and Ernst et al. [10] presented an algorithm especially for short time series. In these models genes in the same cluster were assumed to share similar time domain profile. The temporal relationships were also explored via more complex models, that is, genetic regulatory networks, which can be constructed via more computationally-demanding algorithms, for example, Zhao et al. [11] and Liang et al. [12]. However, in general, the network inference schemes deal only with relatively small-scale networks consisting of less than hundreds of genes. Genome wide analysis is beyond the computational capability of these inference algorithms. Therefore, clustering methods are usually exploited to partition genes, and the obtained subsets of genes serve as further research targets, and more accurate maps of real biological processes are to be recovered.

Based on time-series data, modern spectral density estimation methods have been exploited to identify periodically expressed genes. Assuming the cell cycle signal to be a single sinusoid, Spellman et al. [13] and Whitfield et al. [14] performed a Fourier transformation on the data sampled with different synchronization methods, Wichert et al. [15] applied the traditional periodogram and Fisher's test, while Ahdesmäki et al. [16] implemented a robust periodicity test procedure assuming non-Gaussian noise. The majority of these works dealt with evenly sampled data, and missing data points were usually filled by interpolation in time domain, or the genes were disregarded if there were too many vacancies.

The biological experiments generally output unequally spaced measurements. The change of sampling frequency is due to missing data and the fact that the measurements are usually event driven, that is, more observations are taken when certain biological events occur, and the measurement process is slowed down when the cell remains quiet. Therefore, an analysis based on unevenly sampled data is practically desired and technically more challenging. The harmonics exploited in discrete Fourier transform (DFT) are no longer orthogonal in the presence of uneven sampling. Lomb [17] and Scargle [18] demonstrated that a phase shift suffices to make the sine and cosine terms orthogonal again. The Lomb-Scargle scheme has been exploited in analyzing the budding yeast data set by Glynn et al. [19]. Stoica and Sandgren [20] updated the traditional Capon method to cope with the irregularly sampled data. Notice also that Wang et al. [21] designed the missing-data amplitude and phase estimation (MAPES) approach, which estimated the missing data and spectrum iteratively through the usage of the Expectation Maximization (EM) algorithm. Although Capon and MAPES methods aim to achieve a better spectral resolution than Lomb-Scargle periodogram, for small sample size, the simpler Lomb-Scargle periodogram appears to possess higher accuracy in the presence of real biological data sets [22].

This paper proposes a novel clustering preprocessing procedure which combines the power spectral density analysis with clustering schemes. Given a set of microarray measurements, the power spectral density of each gene is first computed, then the spectral information is fed into the clustering schemes. The members within the same cluster will share similar spectral information, therefore they are supposed to participate in the same temporally regulated biological process. The assumptions underlying this statement rely on the following facts: if two genes X and Y are in the same cluster, their spectral densities are very close to each other; in the time domain, their gene expressions may just differ in their phases. The phases are usually modeled to correspond to different stages of the same biological processes, for example, cell cycle or circadian rhythms. The proposed spectral-density-based clustering actually differentiates the following two cases.

(1) Gene X's expression and Gene Y's expression are uncorrelated in both time and frequency domains.

(2) Gene X and Y expressions are uncorrelated in time domain, but gene X's expression is a time-shifted version of gene Y's expression.

In the traditional clustering schemes, the distances are the same for the above two cases (both assuming large values). However, in the proposed algorithm, the second case is favorable and presents a lower distance. Therefore, by exploiting the proposed algorithm, the genes participating in the same biological process are more likely to be grouped into the same cluster. Lomb-Scargle periodogram serves as the spectral density estimation tool since it is computationally simple and possesses higher accuracy in the presence of unevenly measured and small-size gene expression data sets. The appropriate clustering method is determined based on intense computer simulations. Three major clustering methods: hierarchical, K-means, and self-organizing map (SOM) schemes are tested with different configurations. The spectra and expression-based clusterings are compared with respect to their ability of grouping cell-cycle genes that have been experimentally verified. The differences between clusterings are recorded and compared in terms of information theoretic quantities.

## 2. Methods

This section explains how to apply the Lomb-Scargle periodogram to time-series gene expressions. Next are formulated briefly the three clustering schemes: hierarchical, K-means, and self-organizing map (SOM). Afterward, we discuss how to validate the clusterings and make comparisons between them. The notational convention is as follows: the matrices and vectors are in bold face, and scalars are represented in regular font.

### 2.1. Lomb-Scargle Periodogram

Most spectral analysis methods, for example, Fourier transform and traditional periodogram employed in Spellman et al. [13] and Wichert et al. [15], rely on evenly sampled data, which are projected on orthogonal sine and cosine harmonics. However, real microarray measurements are not evenly observed due to missing data points and changing sampling frequency. The uneven sampling ruins data projection's orthogonality. Lomb [17] found that a phase shift of the sine and cosine functions would restore the orthogonality among harmonics. Scargle [18] complemented Lomb's periodogram by exploiting its distribution. Since then the established Lomb-Scargle periodogram has been exploited in numerous fields and applications, including bioinformatics and genomics (see, e.g., Glynn et al. [19]).

Given  time-series observations , where  stands for the time tag and  denotes the sampled expression of a specific gene, the normalized Lomb-Scargle periodogram for that gene expression at angular frequency  is defined as size(1)

where  and  stand for the mean and variance of the sampled data, respectively, and  is defined as(2)

Let  be the greatest common divisor (gcd) for all intervals , Eyer and Bartholdi [23] proved that the highest frequency to be searched is given by(3)

The number of probing frequencies is denoted by(4)

and the frequency grid can be defined in terms of the following equation:(5)

Notice further that the spectra at the front and rear halves of the frequency grid are symmetric since the microarray experiments output real values.

Lomb-Scargle periodogram represents an efficient solution in estimating the spectra of unevenly sampled data sets. Simulation results also verify its superior performance for biological data with small sample size and various unevenly sampled patterns [22].

### 2.2. Clustering

The obtained Lomb-Scargle power spectral density will be used as input to clustering schemes as an alternative to the original gene expression measurements. Three clustering schemes: Hierachical, K-means, and self-organizing map (SOM) are used for testing this substitution.

#### 2.2.1. Hierarchical Clustering

The hierarchical clustering represents the partitioning procedure that assumes the form of a tree, also known as the dendrogram. The bottom-up algorithm starts in treating each gene as a cluster. Then at each higher level, a new cluster is generated by joining the two closest clusters at the lower level. In order to quantize the distance between two gene profiles, different metrics have been proposed in literature, as enumerated in Table 1.

**Table 1:** Distance metric between two genes' measurements  and

Distance	Formula of	Remarks
Euclidean		is the matrix transpose.
City block		represents sample size, and indexes a specific sample.
Cosine		
Correlation		, are means of vectors and , respectively.

The correlation is the most popular metric and was exploited in Eisen's work [2]. Based on distances between gene expressions, we can further define the distances between two gene clusters, that is, linkage methods, as illustrated by Table 2.

**Table 2:** Distance metric between two clusters  and

Distance	Formula of	Remarks
Single		is defined in Table 1.
Complete		
Average		obtains the size of the cluster.

The single linkage method actually constructs a minimal spanning tree, and it sometimes builds an undesirable long chain. The complete linkage method discourages the chaining effect and in each step increases the cluster diameter as little as possible. However, it assumes that the true clusters are compact. Alternatively, the average linkage method makes a compromise and is usually the preferred method since it poses no assumption on the structure of clusters. The selection of distance metric and linkage method depends on the nature of the real data, and several clustering schemes were proposed to be tested at the same time so that each can capture different aspects of the data. The hierarchical clustering scheme can be formulated in terms of the pseudo code depicted in Algorithm 1. If a specific number of clusters  are desired, only line 3 is needed to be changed by substituting  for .

**Algorithm 1:**Hierarchical clustering algorithm.

1: Input  genes with their expressions or spectral densities;

2: Initialize ; 

3: **while****do**

4:* *;

5:* *Insert , delete  and ;

6:* *Label all existing clusters with integers ;

7:* *

8: **end while**

#### 2.2.2. K-Means Clustering

The K-means clustering divides the genes into  predetermined clusters. It iteratively updates the centroid of each cluster and reassigns each gene to the cluster with the nearest centroid. Different distance metrics, as listed in Table 1, can also be exploited in the K-means clustering scheme. In each iteration, the new centroid might be the median or mean of the cluster members. The K-means clustering can be formulated as Algorithm 2. One of the problems associated with K-means clustering is that the iterations may finally converge to a local suboptimum solution. Therefore, in our simulation we ran the algorithm 5 times and reported the one with the best performance. The K-means clustering method was exploited by Tavazoie et al. [4], which combined the clustering with the motif finding problem.

**Algorithm 2:**K-means clustering algorithm.

1: Input gene expressions or spectral densities, and the desired number of clusters ;

2: Randomly create centroids ;

3: Assign each gene  to the cluster ;

4: **while** members in some clusters change **do**

5:* *compute centroids ;

6:* *assign gene  to cluster ;

7: **end while**

#### 2.2.3. Self-Organizing Map (SOM) Clustering

The self-organizing map method is in essence based on a one-layer neural network, and it is exploited in [3]. Each cluster centroid maps to a node in the two-dimensional lattice. It iteratively updates the centroid of each cluster through competitive learning. At iteration , a randomly selected gene's expression vector  is fed to the learning system, and the centroid which is closest to the coming gene's expression vector is represented in terms of . Then each centroid is updated via(6)

where the function  defines the distance between two nodes indexed by  and  in the two-dimensional lattice. It can be set to 1 if node  is within the neighborhood of node , and 0 otherwise. The function  represents the learning rate function, and it is monotonically decreasing with the increase of  or . The SOM clustering algorithm can be formulated as Algorithm 3.

**Algorithm 3:**SOM clustering algorithm.

1: Input gene expressions or spectral densities, the desired number of clusters , and the number of max iterations ;

2: Randomly create centroids ;

3: Assign each gene  to the cluster ;

4: **for** to **do**

5:* *Randomly select a gene expresssion ;

6:* *Find the point ;

7:* *Update centroids  based on (6);

8: **end for**

9: Assign each gene  to cluster ;

### 2.3. Performance Evaluation Metric

The three clustering schemes with inputs of either gene expressions or spectral densities are to be evaluated in two different ways: how they group time-regulated genes, and whether they are significantly different from each other. Different criteria are defined based on information theoretic quantities.

#### 2.3.1. Validation of Clustering Scheme

Given  genes with their expression or spectral density information , suppose the clustering scheme creates a partition of genes containing  clusters , any two clusters  and  are mutually exclusive (), and all clusters constitute the measured gene expressions (), then the entropy of the clustering can be exploited to measure the information of the clustering(7)

where  measures the size of a cluster. Genes cooperate by participating in the same biological processes, in other words, singleton clusters are not expected to occur frequently in the clustering. Therefore, for a given  the sizes of clusters should be balanced, and the higher the entropy of the clustering, the better the clustering scheme.

The clustering schemes can be validated by their ability to group genes that have been annotated to share similar biological functions or participate in the same biological process. One of the most explored processes is the yeast cell cycle, for which genes have been mostly identified and their interactions have been proposed in the public database [24]. Assume a set of genes, denoted as , has been verified to participate in a specific process, the joint entropy of the clustering and the known set can be represented by(8)

It is desirable that genes with the same functions be integrated in as small number of clusters as possible. Therefore, the smaller the joint entropy, the better the clustering.

A straightforward performance metric combining both the clustering entropy and the joint entropy is defined as the mutual information(9)

where the  is defined similarly as in (7), and it is constant across different clustering schemes. This metric is actually consistent with that proposed in Gibbons and Roth [25], whereby multiple gene attributes were considered. Higher mutual information between the clustering  and the prespecified set  stands for a balanced clustering for all genes while genes of  are more accumulated, in other words, it exhibits better performance.

#### 2.3.2. Difference between Two Clusterings

Two clustering schemes create two different partitions of all the observed genes. A measure of the distance between two clusterings is highly valuable when the two schemes do not show a significant difference in their performance. Various metrics have been proposed to evaluate the difference between two clusterings, for example, Fowlkes and Mallows [26], Rand [27], and more recently Meilă [28]. We accept Meilă's variation of information (VI) metric because it is more discriminative, makes no assumption on the clustering structure, requires no rescaling, neither does it depend on the sample size.

Assume two different schemes produce two clusterings  and , respectively, then the mutual information between these two clusterings is represented by(10)

Then, the variation of information (VI) is defined as(11)

VI is upper bounded by . It is zero if and only if the two clusterings are exactly the same. The greater the variation of information, the larger the difference between the two clusterings.

## 3. Results

The performance of the proposed power spectrum-based scheme is illustrated through comparisons with three traditional expression-based clustering schemes: Hierarchical, K-means, and self-organizing map (SOM). The comparisons are divided into two parts. In the first part, we evaluate their ability to group the cell-cycle involved genes, while the second part is devoted to illustrate the fact that the proposed schemes construct clusters that are significantly different from those created by the traditional schemes.

### 3.1. Clustering Performance Evaluation

These simulations were performed on the cdc15 data set published by Spellman et al. [13], which contained 24 time-series expression measurements of 6178 yeast genes. The hierarchical, K-means, and self-organizing map (SOM) clustering schemes were simulated having as inputs the computed spectral densities and the original expression data. The hierarchical and K-means clustering were configured with different distance and linkage methods, which are defined in Tables 1 and 2, respectively. The simulations were executed until up to 200 clusters were created.

Cell cycle has served as a research target in molecular biology for a long time since it plays a crucial rule in cell division, and medically it underlies the development of cancer. Experimentally 109 genes have been verified to participate in the cell-cycle process, and their interactions were recorded in the public database KEGG [24]. Among them 104 genes were reported in Spellman's data set. The simulations tested how these genes were clustered with other genes. Intuitively, the more integrated are these 104 genes, the better is the clustering scheme. On the other hand, it is hoped that the size of the cluster is relatively balanced, and there should not be many singleton clusters (clusters containing only one gene).

The clustering performance is represented by an information theoretic quantity, that is, mutual information, which is defined between the obtained partition of all measured genes and the set of 104 genes. Higher mutual information indicates that the 104 cell-cycle genes are closely integrated into only a few clusters, and most clusters are balanced in size. In other words, with the same number of clusters, the higher the mutual information, the better the performance.

The proposed strategy is surely not constrained to detect cell cycle genes. However we have to confine our discussion to cell cycle here because the available data set is right for the purpose of cell cycle research. Besides, the cell cycle genes have been identified for a relatively long time with high confidence.

**Figure 1:**
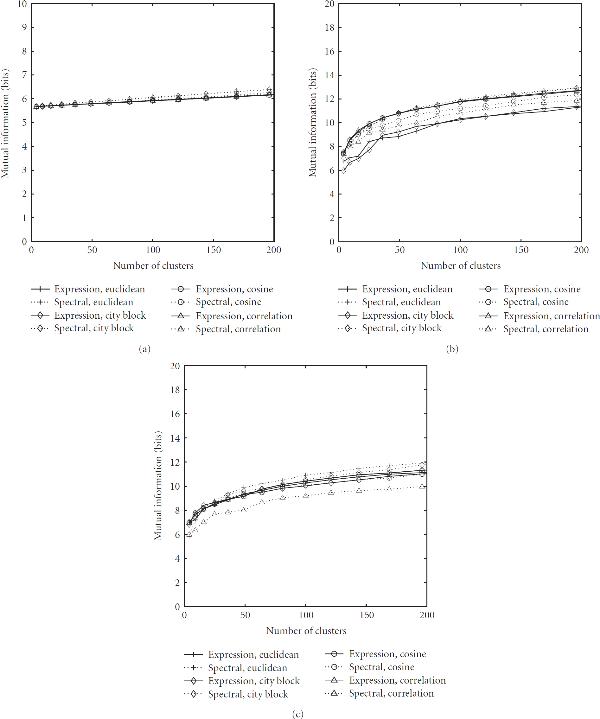
**Performance of hierarchical clustering: (a) single linkage, (b) complete linkage, and (c) average linkage**. The solid curves represent the clusterings based on original gene expressions while the dotted curves stand for clusterings based on spectral densities.

The simulation results for hierarchical clustering are illustrated in Figure 1. Each subplot is associated with a linkage method. Figure 1(a) demonstrates the performance for the single linkage method. The dotted curves represent schemes clustering spectral densities while the solid curves denote schemes clustering original gene expressions. The mutual information goes up nearly linearly when the number of clusters increases. Actually, when we delved into the generated clusters, it was found that most clusters were singletons. The chaining effect took place, and the single linkage method is not a good candidate for the purpose of clustering gene expression measurements. Spectral density-based methods were all better than their traditional counterparts, which performed clustering on the original gene expression data. Among all, the Euclidean method clustering spectral densities achieved the best performance.

Figure 1(b) shows the results for the complete linkage method of the hierarchical clustering. Each cluster actually represents a complete subgraph. The complete linkage method discourages the chaining effect to occur in the single linkage method. The performance of spectral density-based clusterings is lower bounded by the worst performances of the traditional gene expression-based clusterings. For the gene expression-based clustering, the correlation and cosine approaches are better than the Euclidean and city-block approaches, while for the spectral density clustering, the Euclidean and city-block approaches exhibit the best performance.

Figure 1(c) plots the results for the average linkage method of the hierarchical clustering. The average linkage is the most widely deployed method since it makes a compromise between the single and the complete methods, and it does not assume any structure on the underlying data. However, in the presence of real gene expression data, it is not as good as the complete linkage method. Different distance metrics differ in terms of their ability to group the involved cell-cycle genes. For clustering expression data, the cosine and correlation approaches still achieve the best performance, but they exhibit poorer performance than the spectra-based Euclidean and city-block methods.

**Figure 2:**
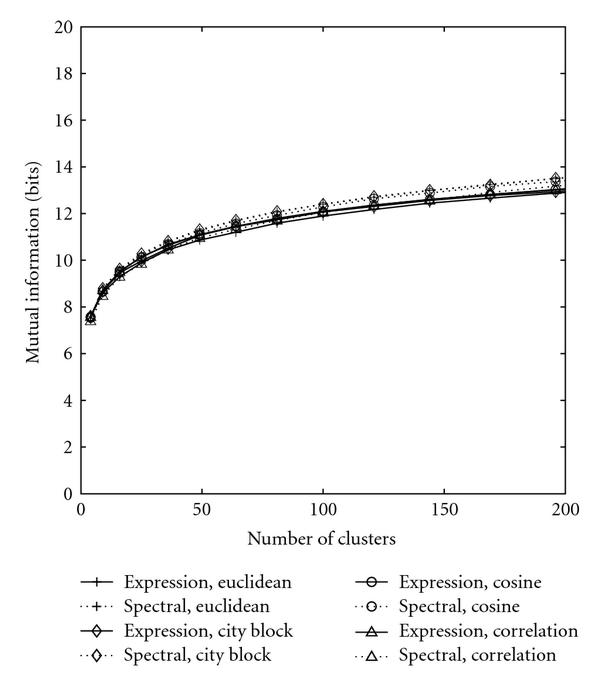
**Performance of K-means clustering**. The solid curves represent the clusterings based on original gene expressions while the dotted curves stand for clusterings based on spectral densities.

Configured also with various distance metrics, the K-means algorithm was applied on both the spectral and original gene expression data. To avoid converging to local suboptimal solutions, all K-means clustering schemes were executed 5 times, and the best performance was reported. For clustering expression data, the correlation and cosine approaches are still the best choices while for spectra-based schemes, the Euclidean and city-block approaches still exceed the other schemes (see Figure 2).

**Figure 3:**
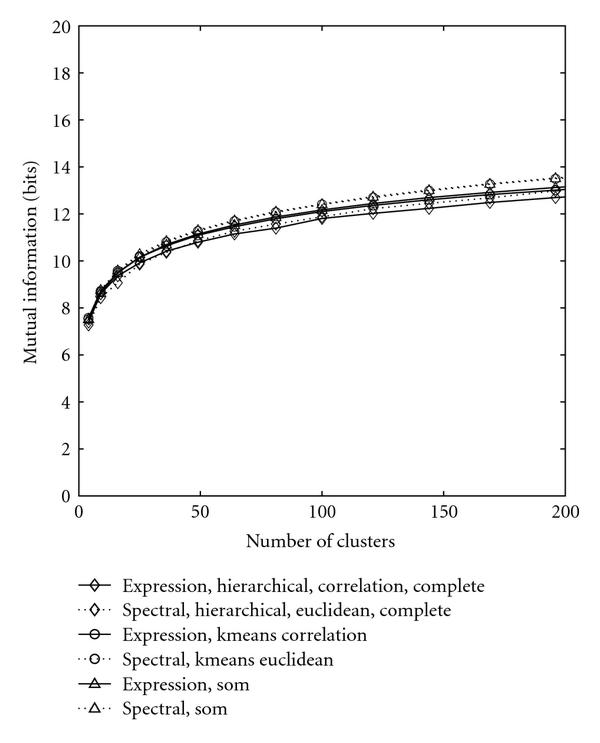
**Performance of hierarchical, K-means, and SOM**. The comparison is performed across the complete linkage of hierarchical, K-means, and SOM. The solid curves represent the clustering based on original gene expression data while the dotted curves stand for clustering based on spectral data.

Figure 3 compares the performance of hierarchical and K-means clustering schemes with that of SOM. The best schemes of hierarchical and K-means were displayed. It turns out that SOM is the best performing scheme, K-means locates in the middle, whereas the hierarchical clustering is the worst, although the discrepancy looks not significant. Among all schemes, the spectral density-based SOM achieves the best performance. Although the discrepancy between the best spectral-based clustering and the best gene expression-based clustering is not obvious, they actually create significantly different clusters. This difference can be captured by the distance metric between clusterings.

The inferior performance of correlation and cosine metrics with spectra input is partially due to the flat spectra for those genes with no time-regulated patterns. The flat spectrum in the denominator will cause the distance metrics to be highly biased. It is also worthwhile to note that in literature other distance metrics have been proposed, for example, coherence [29] and mutual information [30]. However, these metrics involve the estimation of joint distribution, which usually requires large sample sizes. Such a requirement cannot be satisfied in general by the microarray experiments. Extra normalization of the spectrum can be performed, but simulation shows that it does not provide a significant or consistent improvement.

### 3.2. Distance between Clusterings

A testing of the distance between spectra-based and gene expression-based clusterings also reveals the value of the proposed scheme. The variation of information metric approach, proposed by Meilă [28], is exploited to measure the difference between the two clusterings. The basic principle resumes to: the higher the variation of information, the greater the difference.

**Figure 4:**
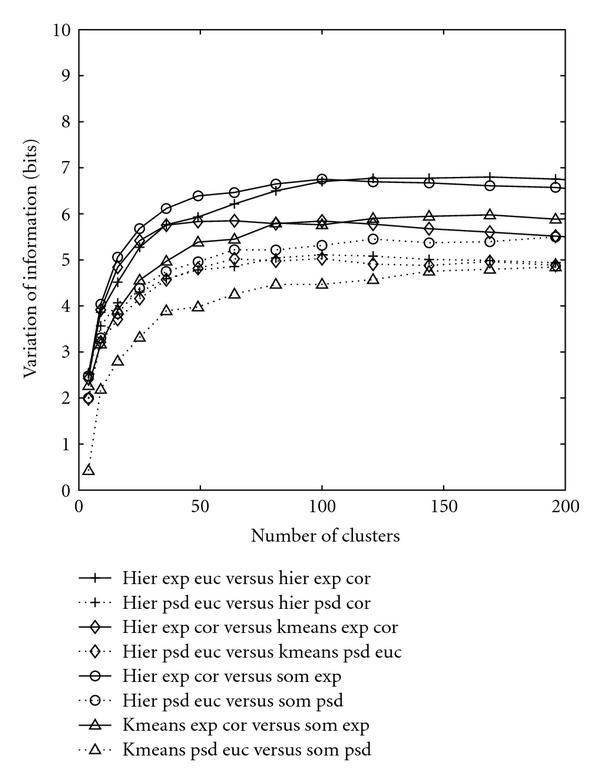
**Distance between the two clusterings created by different methods with the same input**. Only the complete linkage for the hierarchical clustering is considered. The solid curves represent the clustering based on original gene expression data while the dotted curves stand for clustering based on spectral densities. Abbreviations are exploited for the conciseness of labels as follows: hier (hierarchical clustering), euc (Euclidean), cor (correlation), psd (power spectral density), exp (expression data).

Figure 4 demonstrates the distance between the two clusterings with the same input, either computed using spectral densities or measured based on gene expressions. For the hierarchical clustering, only the complete linkage method is considered since it possesses the best performance in terms of grouping the known cell-cycle genes. The complete set of distances between any two schemes is depicted in the additional File 1 [31]. Figure 4 conserves only the salient general patterns for conciseness. For hierarchical clustering of gene expression data, the correlation and Euclidean schemes differ more, and the distance between these two is the highest curve when the number of clusters is greater than 120. The distance between the correlation and Euclidean hierarchical clusterings is even much larger than the distance between the clusterings created by the hierarchical scheme and K-means or SOM. However, when clustering spectral densities, all schemes display quite similar patterns and exhibit closely located performances. This means that clustering spectral densities is stable across different clustering schemes.

**Figure 5:**
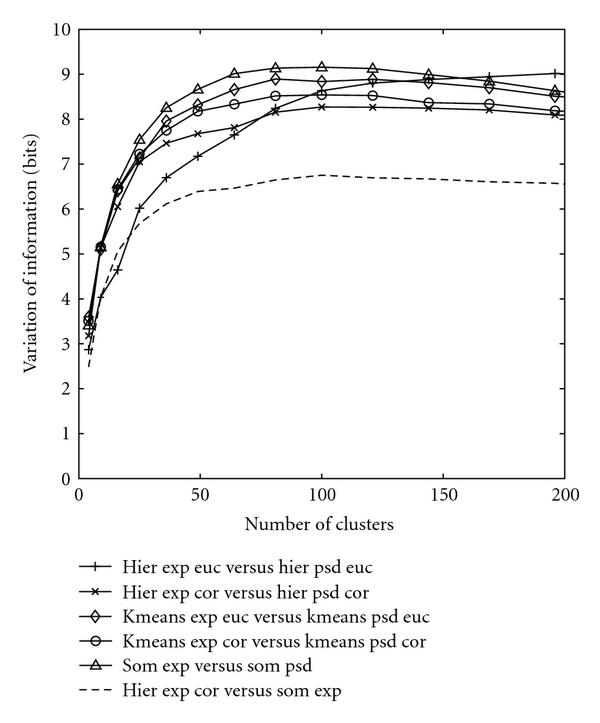
**Distance between two clusterings created by the same method assuming different inputs**. The comparison is performed across the complete linkage of hierarchical, K-means, and SOM. The dashed curve is provided with the purpose of reference. Abbreviations are exploited for the conciseness of labels as follows: hier (hierarchical clustering), euc (Euclidean), cor (correlation), psd (power spectral density), exp (expression data).

Figure 5 compares the same clustering methods assuming different inputs. Comparing with the scale of Figure 4, the distance between different clusterings with the same input is much smaller than the distance between clusterings that assume different input types. The distance between any two schemes that assume the same input is below 7 bits when the number of clusters is ranging from 0 to 200, as shown in Figure 4 or the dashed curve in Figure 5, while the distance between the clusterings created by the same scheme assuming two different input types is above 8 bits when the number of clusters is ranging from 100 to 200. This shows that changing the input type from gene expression to spectral density has produced a significant different clustering scheme. For the complete plots of the distance between clusterings produced by various schemes assuming different input types, please refer to the additional File 2 [31].

## 4. Conclusion

A novel clustering preprocessing strategy is proposed to combine the traditional clustering schemes with power spectral analysis of time-series gene expression measurements. The simulation results corroborate that the proposed approach achieves a better clustering for hierarchical, K-means, and self-organizing map (SOM) in most cases. Besides, it constructs a significantly different partition relative to traditional clustering strategies. When deploying the hierarchical or K-means clustering methods based on the spectral density, the Euclidean and city-block distance metrics appear to be more appealing than the cosine or correlation distance metrics. The proposed novel algorithm is valuable since it provides additional information about temporal regulated genetic processes, for example, cell cycle.

## References

[B1] Simon I Siegfried Z Ernst J Bar-Joseph Z Combined static and dynamic analysis for determining the quality of time-series expression profiles Nature Biotechnology 2005 23 12 1503 1508 10.1038/nbt1164 16333294

[B2] Eisen MB Spellman PT Brown PO Botstein D Cluster analysis and display of genome-wide expression patterns Proceedings of the National Academy of Sciences of the United States of America 1998 95 25 14863 14868 10.1073/pnas.95.25.14863 9843981 PMC24541

[B3] Tamayo P Slonim D Mesirov J Interpreting patterns of gene expression with self-organizing maps: methods and application to hematopoietic differentiation Proceedings of the National Academy of Sciences of the United States of America 1999 96 6 2907 2912 10.1073/pnas.96.6.2907 10077610 PMC15868

[B4] Tavazoie S Hughes JD Campbell MJ Cho RJ Church GM Systematic determination of genetic network architecture Nature Genetics 1999 22 3 281 285 10.1038/10343 10391217

[B5] Zhou X Wang X Dougherty ER Russ D Suh E Gene clustering based on clusterwide mutual information Journal of Computational Biology 2004 11 1 147 161 10.1089/106652704773416939 15072693

[B6] Giurcăneanu CD Tăbuş I Astola J Ollila J Vihinen M Fast iterative gene clustering based on information theoretic criteria for selecting the cluster structure Journal of Computational Biology 2004 11 4 660 682 15579237 10.1089/1066527041887285

[B7] D'Haeseleer P How does gene expression clustering work? Nature Biotechnology 2005 23 12 1499 1501 10.1038/nbt1205-1499 16333293

[B8] Ramoni MF Sebastiani P Kohane IS Cluster analysis of gene expression dynamics Proceedings of the National Academy of Sciences of the United States of America 2002 99 14 9121 9126 10.1073/pnas.132656399 12082179 PMC123104

[B9] Tabus I Astola J Clustering the non-uniformly sampled time series of gene expression data Proceedings of the International Symposium on Signal Processing and Applications (ISSPA '03), Paris, France, July 2003 2 61 64

[B10] Ernst J Nau GJ Bar-Joseph Z Clustering short time series gene expression data Bioinformatics 2005 21 supplement 1 i159 i168 15961453 10.1093/bioinformatics/bti1022

[B11] Zhao W Serpedin E Dougherty ER Inferring gene regulatory networks from time series data using the minimum description length principle Bioinformatics 2006 22 17 2129 2135 10.1093/bioinformatics/btl364 16845143

[B12] Liang S Fuhrman S Somogyi R Reveal, a general reverse engineering algorithm for inference of genetic network architectures Proceedings of the Pacific Symposium on Biocomputing, Maui, Hawaii, USA, January 1998 3 18 29 9697168

[B13] Spellman PT Sherlock G Zhang MQ Comprehensive identification of cell cycle-regulated genes of the yeast Saccharomyces cerevisiae by microarray hybridization Molecular Biology of the Cell 1998 9 12 3273 3297 9843569 10.1091/mbc.9.12.3273 PMC25624

[B14] Whitfield ML Sherlock G Saldanha AJ Identification of genes periodically expressed in the human cell cycle and their expression in tumors Molecular Biology of the Cell 2002 13 6 1977 2000 10.1091/mbc.02-02-0030. 12058064 PMC117619

[B15] Wichert S Fonkianos K Strimmer K Identifying periodically expressed trascripts in microarry time series data Bioinformatics 2004 20 1 5 20 10.1093/bioinformatics/btg364 14693803

[B16] Ahdesmäki M Lähdesmäki H Pearson R Huttunen H Yli-Harja O Robust detection of periodic time series measured from biological systems BMC Bioinformatics 2005 6, article 117 1 18 15892890 10.1186/1471-2105-6-117 PMC1168888

[B17] Lomb NR Least-squares frequency analysis of unequally spaced data Astrophysics and Space Science 1976 39 2 447 462 10.1007/BF00648343

[B18] Scargle JD Studies in astronomical time series analysis—II. Statistical aspects of spectral analysis of unevenly spaced data The Astrophysics Journal 1982 263 99 835 853

[B19] Glynn EF Chen J Mushegian AR Detecting periodic patterns in unevenly spaced gene expression time series using Lomb-Scargle periodograms Bioinformatics 2006 22 3 310 316 10.1093/bioinformatics/bti789 16303799

[B20] Stoica P Sandgren N Spectral analysis of irregularly-sampled data: paralleling the regularly-sampled data approaches Digital Signal Processing 2006 16 6 712 734 10.1016/j.dsp.2006.08.012

[B21] Wang Y Stoica P Li J Marzetta TL Nonparametric spectral analysis with missing data via the EM algorithm Digital Signal Processing 2005 15 2 191 206 10.1016/j.dsp.2004.10.004

[B22] Zhao W Agyepong K Serpedin E Dougherty ER Detecting periodic genes from irregularly sampled gene expressions: a comparison study EURASIP Journal on Bioinformatics and Systems Biology 2008 2008 8 10.1155/2008/769293 PMC3171399 18584052

[B23] Eyer L Bartholdi P Variable stars: which Nyquist frequency? Astronomy and Astrophysics 1999 135 1 1 3

[B24] KEGG Yeast Cell Cycle Pathway http://www.genome.ad.jp/kegg/pathway/sce/sce04111.html

[B25] Gibbons FD Roth FP Judging the quality of gene expression-based clustering methods using gene annotation Genome Research 2002 12 10 1574 1581 10.1101/gr.397002 12368250 PMC187526

[B26] Fowlkes E Mallows C A method for comparing two hierarchical clusterings Journal of the American Statistical Association 1983 78 383 553 569 10.2307/2288117

[B27] Rand WM Objective criteria for the evaluation of clustering methods Journal of the American Statistical Association 1971 66 336 846 850 10.2307/2284239

[B28] Meilă M Comparing clusterings—an information based distance Journal of Multivariate Analysis 2007 98 5 873 895 10.1016/j.jmva.2006.11.013

[B29] Butte AJ Bao L Reis BY Watkins TW Kohane IS Comparing the similarity of time-series gene expression using signal processing metrics Journal of Biomedical Informatics 2001 34 6 396 405 10.1006/jbin.2002.1037 12198759

[B30] Brillinger DR Second-order moments and mutual information in the analysis of time series Recent Advances in Statistical Methods 2002 Imperial College Press, London, UK 64 76

[B31] Supplementary Materials http://www.ece.tamu.edu/~wtzhao/EurasipBSBClutering.htm

